# Society Meetings

**Published:** 1901-07

**Authors:** 


### SOCIETY MEETINGS.

Dr Henry L. Elsner, president of the Medical Society of the State of New York, has announced the appointment of the business committee for the ensuing year, consisting of Dr. Nathan Jacobson, chairman, 430 S. Salina Street, Syracuse; Dr. George Ryerson Fowler, 301 DeKalb Avenue, Brooklyn, and Dr. William C. Krauss, 371 Delaware Avenue, Buffalo. All letters and inquiries pertaining to papers and scientific communications for the semiannual meeting to be held in New York City, October 15 and 16, 1901, and the annual meeting to be held in Albany, January, 1902, should be addressed to the chairman.

The congress on tuberculosis, which is to be held in London, July 23-26, 1901, promises to be a pronounced success, alike as regards attendance and the importance of the contributions that will be made to medical science. Professor Koch, of Berlin, and Dr. Roux, of Paris, are among the delegates who are expected from foreign countries, and the possibility of devising means for the stamping out of tuberculosis will be discussed from the points of view, not only of the doctor, but also of the sanitarian, the municipal ruler and the statesman. For the convenience of physicians from this country who intend being present an excursion has been arranged at reduced rates. The steamer will leave New York on July 10th and sufficient time will be allowed the party to visit Paris and also to attend the meeting of the British Medical Association, which will be held at Cheltenham in the last week of July. An entertainment fund of over $15,000 has been raised in connection with the tuberculosis congress, among the subscribers being many well known members of the British nobility and gentry, the corporations of the principal cities, the Rothschilds and other representatives of financial interests of Europe, and a large number of commercial houses, including Parke, Davis & Co. and some other houses interested in therapeutics. The arrangements for the trip are in the hands of Gaze & Sons, 113 Broadway, New York.

---

THE Buffalo Academy of Medicine held meetings during the month of June, 1901, as follows:

*Section on Surgery.*—Tuesday evening, June 4th. Program: Recent development in the cancer problem, H. R. Gaylord; demonstration with stereopticon.

*Annual Meeting.*—Tuesday evening, June 11th. Program: Election of officers. Reports of officers. An address by the retiring president, Marcell Hartwig.

*Section on Pathology.*—Tuesday evening, June 18th. Program: A case of pseudo-leukemia apparently developing into true lymphatic leukemia, accompanied by leukemic lesions and pigmentations, culminating in streptococcus infection, Grover W. Wende; discussion by Charles G. Stockton, Herbert U. Williams, Albert E. Woehnert and Irving P. Lyon.

*Quarterly Meeting.*—Tuesday evening, June 25th. Program: Acute respiratory failure occurring during the first few days of life; not due to asphyxia or congenital heart disease, Irving M. Snow. Discussed by H. D. Ingraham and A. J. Colton.

THE American Congress of Tuberculosis held an interesting meeting in New York, May 15 and 16, 1901, under the presidency of the Hon. Clark Bell of that city. The officers elected were as follows: Honorary president, A. N. Bell, Brooklyn, N. Y.; president, Henry D. Holton, secretary state board of health, Brattleboro, Vt.; secretary and treasurer, Clark Bell, Esq., City of New York. Dr. John H. Pryor, of Buffalo, was elected one of the vice-presidents.

---

THE Röntgen Society of the United States will hold its second annual meeting at Buffalo in the University building, September 10 and 11, 1901, under the presidency of Dr. Heber Robarts, of St. Louis. The secretary is Dr. J. Rudis-Jicinsky, of Cedar Rapids, Iowa: Committee on arrangements, Edgar B. Stevens, chairman, 688 Seventh street, Buffalo; Roger Cook Adams, Secretary, Drawer 963, Buffalo; Dr. Elmer E. Starr, 523 Delaware Avenue, Buffalo; Dr. Renwick R. Ross, 100 High Street, Buffalo; Dr. James W. Putnam, 525 Delaware Avenue, Buffalo.

---

THE American Laryngological, Rhinological and Otological Society, eastern section, held its annual meeting June 21 and 22, 1901, at the Library Building, under the presidency of Dr. W. Scott Renner, of Buffalo. A number of prominent laryngologists were present from important cities east of the Alleganies and an interesting program was disposed of. On Friday Dr. Renner entertained the society at luncheon at the Saturn Club, together with about thirty Buffalo physicians, whom he invited to meet the members.

---

THE fourth district branch, New York State Medical Association, was held at Buffalo, May 31, 1901, under the presidency of Dr. William H. Thornton. The officers chosen for the ensuing year are: President, C. A. Wall, of Buffalo; vice-president, J. W. Morris, of Jamestown; secretary, Bernard Cohen, of Buffalo; treasurer, W. Irving Thornton, of Buffalo.

---

THE Medical Society of the County of Erie held its eightieth semiannual meeting at Buffalo, June 11, 1901, under the presidency of Dr. William C. Phelps. The meeting was an exceptionally well attended and interesting one. The scientific program included a paper by Dr. Henry L. Elsner, of Syracuse, president of the Medical Society of the State of New York, entitled, Malignant endocarditis; and one by Henry R. Hopkins, of Buffalo, entitled, The fan system of heating and ventilating and the air supply of certain Buffalo schools.

---

THE American Association of Obstetricians and Gynecologists will hold its fourteenth annual meeting at the Hotel Hollenden, Cleveland, O., Tuesday, Wednesday and Thursday, September 17, 18 and 19, 1901, under the presidency of Dr. William E. B. Davis, of Birmingham, Ala. The committee of arrangements is composed of Dr. M. Rosenwasser, 722 Woodland Avenue and Dr. William H. Humiston, 122 Euclid Avenue, Cleveland, either of whom may be addressed concerning rooms or other local information regarding the meeting.

---

THE American Public Health Association will hold its twenty-ninth annual meeting at Buffalo, Monday, Tuesday and Wednesday, and Thursday and Friday, September 16-20, 1901, under the presidency of Dr. Benjamin Lee, of Philadelphia. Dr. Henry R. Hopkins, 433 Franklin Street, Buffalo, is the chairman of the committee of arrangements, who may be addressed concerning any local information pertaining to the meeting.

---

THE American Gynecological Society held its twenty-sixth annual meeting at Chicago, May 30-June 1, 1901, under the presidency of Dr. Ely Van de Warker, of Syracuse. The meeting was an extremely interesting one and among many papers of interest one, entitled, Extirpation of the bladder in the female for cancer, by Matthew D. Mann, of Buffalo, deserves special mention. Dr. Mann reported two operations for this condition which we believe are the first made in this country. Dr. S. C. Gordon, of Portland, Maine, was elected president for the ensuing year.

---

THE Medical Association of Central New York will hold its thirty-fourth annual meeting at Buffalo, August 27 and 28, 1901, at the Library Building, under the presidency of Dr. Lucien Howe, 183 Delaware Avenue, Buffalo. Dr. Charles P. Van der Beek, of 16 Gibbs Street, Rochester, is the secretary.

